# Ambulatory Endoscopic Thyroidectomy *via* a Chest-Breast Approach Has an Acceptable Safety Profile for Thyroid Nodule

**DOI:** 10.3389/fendo.2021.795627

**Published:** 2021-12-20

**Authors:** Zeyu Zhang, Fada Xia, Xinying Li

**Affiliations:** Department of Thyroid Surgery, Xiangya Hospital, Central South University, Changsha, China

**Keywords:** ambulatory surgery, endoscopic thyroidectomy, thyroid nodule, postoperative complication, mental health

## Abstract

**Introduction:**

With the growing esthetic requirements, endoscopic thyroidectomy develops rapidly and is widely accepted by practitioners and patients to avoid the neck scar caused by open thyroidectomy. Although ambulatory open thyroidectomy is adopted by multiple medical centers, the safety and potential of ambulatory endoscopic thyroidectomy via a chest-breast approach (ETCBA) is poorly investigated.

**Material and Methods:**

Patients with thyroid nodules who received conventional or ambulatory ETCBA at Xiangya hospital, Central South University from January 2017 to June 2020 were retrospectively included. The incidence of postoperative complications, 30-days readmission rate, financial cost, duration of hospitalization, mental health were mainly investigated.

**Results:**

A total of 260 patients were included with 206 (79.2%) suffering from thyroid carcinoma, while 159 of 260 received ambulatory ETCBA. There was no statistically significant difference in the incidence of postoperative complications (P=0.249) or 30-days readmission rate (P=1.000). In addition, The mean economic cost of the ambulatory group had a 29.5% reduction compared with the conventional group (P<0.001). Meanwhile, the duration of hospitalization of the ambulatory group was also significantly shorter than the conventional group (P<0.001). Patients received ambulatory ETCBA showed a higher level of anxiety (P=0.041) and stress (P=0.016). Subgroup analyses showed consistent results among patients with thyroid cancer with a 12.9% higher complication incidence than the conventional ETCBA (P=0.068).

**Conclusion:**

Ambulatory ETCBA is as safe as conventional ETCBA for selective patients with thyroid nodules or thyroid cancer, however with significant economic benefits and shorter duration of hospitalization. Extra attention should be paid to manage the anxiety and stress of patients who received ambulatory ETCBA.

## Introduction

Thyroid nodule is a very common disease and often discovered by accident mainly because of the asymptomatic feature (1).Although there is an increasing incidence of thyroid malignancy, most of thyroid nodules are benign without the need for invasive therapies (2). For patients who reach the indication of surgery, a traditional open thyroidectomy is the primary choice. However, with the growing esthetic requirements, endoscopic thyroidectomy develops rapidly and is widely accepted by practitioners and patients to avoid the neck scar caused by open thyroidectomy (3). Increasing evidences have shown that endoscopic thyroidectomy has the same efficacy and safety profiles as the open thyroidectomy (4–6).

Ambulatory surgery has become more and more common in recent years, offering various advantages (7). At present, ambulatory open thyroidectomy is adopted by multiple medical centers, showing no difference in readmissions and complications compared with conventional thyroidectomy (8). Moreover, with the development of instruments and techniques, many surgical procedures with a higher complexity are subsequently allowed to be performed with an ambulatory management (9).

Endoscopic thyroidectomy is usually carried out with multiple-day hospitalization, while few medical centers perform ambulatory endoscopic thyroidectomy. In addition, as far as we know, there is no study focusing on the potential of ambulatory endoscopic thyroidectomy. In our medical center, both conventional and ambulatory endoscopic thyroidectomy via a chest-breast approach (ETCBA) is routinely performed. Thus, in this study, we retrospectively evaluated the efficacy and safety of ambulatory ETCBA.

## Material and Methods

### Study Cohort

Patients with thyroid nodules who received conventional or ambulatory ETCBA at Xiangya hospital, Central South University from January 2017 to June 2020 were retrospectively included. The exclusion criteria were as follows: (1) signs of extrathyroidal tissue invasion; (2) previously received neck surgery or radiation; (3) suffering from infectious diseases or parathyroid disease; (4) lack of essential clinical data, including results of ultrasound, parathyroid hormone, complication data, and assessment of mental health. Patients were divided into conventional group and ambulatory group according to the surgery they received. This study was approved by the Institutional Ethics Committee of Xiangya Hospital, Central South University (No.202011960) and met the guidelines of governmental ethics agency in accordance with the precepts established by the Helsinki Declaration.

### Surgical Procedures and Postoperative Management

The preoperative examination, surgeons, and procedures of ETCBA were identical between the two groups. The operative position and the location of operating and observing ports were as same as in the previous report (10). Patients with benign nodules would receive a thyroidectomy, while a central lymph node dissection was additionally performed among patients with malignant nodules. For patients with an undetermined result of fine-needle aspiration (FNA) biopsy, intraoperative frozen section histological examination was performed to determine the further procedures. Generally, ETCBA was not administrated for patients with lateral lymph node metastasis detected by preoperative examinations. However, lateral lymph node dissection was still performed endoscopically when suspicious lateral lymph node metastasis was intraoperatively detected. Patient status and vital signs were carefully monitored after surgery, and discharge assessment included vital signs, consciousness, airway status and sound, incision condition, and serum calcium. Calcium and analgesics were given as appropriate. The removal of the drainage tube and dressing change were performed the next morning. Patients in the conventional group were usually discharged on the third day after ETCBA, while the ambulatory group within 24 hours after ETCBA.

### Outcomes and Follow-Up

Medical records and pathological reports were investigated for clinical data and patient characteristics. Every patient was followed up with examinations including ultrasound, thyroid function, and parathyroid hormone (PTH) every 3-6 months after discharge. The outcomes of this study were mainly incidence of postoperative complications, 30-days readmission rate, financial cost, duration of hospitalization. Hypoparathyroidism was recognized as PTH level <15 pg/ml, and hypoparathyroidism and the voice change recovered in six months was considered as transient. An independent surgeon was assigned to record these data.

Furthermore, the mental health of patients was also investigated within one month, using the Depression Anxiety Stress Scales-21 (DASS-21), a screening tool to measure three comorbid depression, anxiety and stress disorders (**Supplement Table 1**) (7).

### Statistical Analysis

SPSS 23.0 was used for statistical analyses. The continuous variables were expressed as mean ± standard deviation and analyzed using the independent-sample t-test or Mann-Whitney U test according to its distribution pattern. Categorical variables were expressed as frequency (percentage) and analyzed using Chi-square or Fisher exact test as appropriate. An independent analyst was assigned to assess the statistics, and statistical significance was recognized with P < 0.05.

## Results

### Patient and Tumor Characteristics

Among a total of 260 included patients, 159 received ambulatory ETCBA, while 101 received conventional one (**Table 1**). The mean age of the two groups was 35.74 ± 8.26 and 34.93 ± 9.13 years, and the patients who received ETCBA were mostly female (92.7%). There was no difference in ASA classification between the two groups (P=0.094). 125 (78.6%) and 81 (80.2%) patients were diagnosed as malignancy in the ambulatory group and conventional group, respectively. 56 (21.5%) patients suffered from multiple lesions. The largest tumor size was 1.22 ± 1.19 cm and 1.25 ± 1.31 cm in ambulatory group and conventional group, respectively. Moreover, no difference was shown in surgical procedures between the two groups (P=0.246), indicating an identical degree of difficulty of surgery. Overall, there was no difference in the patient and tumor characteristics between two groups.

**Table 1:** Patients and nodules characteristics.

Characteristic	Ambulatory group (n=159)	Conventional group (n=101)	*P*
Age (years)	35.74 ± 8.26	34.93 ± 9.13	0.459
Gender			0.090
Female	151 (95.0)	90 (89.1)	
Male	8 (5.0)	11 (10.9)	
Ethnicity			
Asian	159 (100.0)	101 (100.0)	1.000
ASA classification			0.094
I	61 (38.4)	50 (49.5)	
II	98 (61.6)	51 (50.5)	
Malignant nodules	125 (78.6)	81 (80.2)	0.876
Number of lesion			0.878
Single	124 (78.0)	80 (79.2)	
Multiple	35 (22.0)	21 (20.8)	
Largest tumor size (cm)	1.22 ± 1.19	1.25 ± 1.31	0.840
Surgical procedure			0.246
Unilateral thyroidectomy	27 (17.0)	13 (12.9)	
Unilateral thyroidectomy with CLND	62 (39.0)	49 (48.5)	
Bilateral thyroidectomy	5 (3.1)	7 (6.9)	
Bilateral thyroidectomy with CLND	62 (39.0)	31 (30.7)	
Thyroidectomy with LLND	3 (1.9)	1 (1.0)	

Data are expressed as mean ± standard deviation or n (%). ASA, American Society of Anaesthesiology; CLND, central lymph node dissection; LLND, lateral lymph node dissection.

### Surgical Outcomes and Prognosis

As shown in **Table 2**, surgical outcomes and patient prognosis were compared between ambulatory and conventional ETCBA. No difference was shown in the duration of surgery and blood loss. The ambulatory group showed a higher incidence of complications (30.8%) than the conventional group (23.8%), however with no statistically significant difference. Notably, all four patients who suffered from permanent hypoparathyroidism were in the ambulatory group. Two patients failed in the ambulatory management due to seroma or hematoma, and were transferred to the conventional ward for surveillance without re-operation. Furthermore, only one patient in the ambulatory group was readmitted due to hypocalcemia. Till the last follow-up, one patient with a malignant nodule suffered from a recurrence in the ambulatory group and conventional group, respectively. There was no patient suffering from re-operation or death in this study.

**Table 2:** Surgical outcomes and prognosis.

Characteristic	Ambulatory group (n=159)	Conventional group (n=101)	*P*
Duration of surgery (min)	186.19 ± 31.77	183.78 ± 30.12	0.544
Blood loss (ml)	18.79 ± 10.79	18.84 ± 8.78	0.964
Postoperative complications	49 (30.8)	24 (23.8)	0.258
Transient hypoparathyroidism	39 (24.5)	20 (19.8)	
Permanent hypoparathyroidism	4 (2.5)	0 (0.0)	
Hypoglycemia	0 (0.0)	1 (1.0)	
Transient voice change	5 (3.1)	2 (2.0)	
Seroma	1 (0.6)	0 (0.0)	
Pulmonary infection	0 (0.0)	1 (1.0)	
Hematoma	1 (0.6)	0 (0.0)	
Transfer to the conventional ward	2 (1.3)	–	–
30-days readmission	1 (0.6)	0 (0.0)	1.000
Recurrence of malignancy	1 (0.6)	1 (1.0)	0.627

Data are expressed as mean ± standard deviation or n (%).

### Economic Cost and Duration of Hospitalization

Economic cost and duration of hospitalization were also investigated (**Table 3**). The mean economic cost of the ambulatory group had a 29.5% reduction compared with the conventional group. Meanwhile, the duration of hospitalization of the ambulatory group was also significantly shorter than the conventional group.

**Table 3:** Economic cost and duration of hospitalization.

Characteristic	Ambulatory group (n=159)	Conventional group (n=101)	*P*
Economic cost (yuan)	14278.57 ± 2194.22	20261.26 ± 4399.18	<0.001
Duration of hospitalization (day)	1.03 ± 0.29	5.51 ± 1.43	<0.001

Data are expressed as mean ± standard deviation.

### Mental Health

**Table 4** showed the results of DASS-21. While there was no significant difference in the level of depression (P=0.731), higher levels of anxiety (P=0.041) and stress (P=0.016) were shown in the ambulatory group than the conventional group.

**Table 4:** Results of DASS-21.

Characteristic	Ambulatory group (n=159)	Conventional group (n=101)	*P*
Depression	3.77 ± 2.06	3.86 ± 1.91	0.731
Anxiety	7.79 ± 2.55	7.12 ± 2.64	0.041
Stress	6.91 ± 2.61	6.07 ± 2.89	0.016

Data are expressed as mean ± standard deviation.

### Subgroup Analyses for Patients With Thyroid Cancer

To further investigate the role of ambulatory ETCBA in patients with thyroid cancer, subgroup analyses were performed excluding patients with benign nodules (**Table 5**). The difference between two groups remained insignificant in duration of surgery, blood loss, 30-day readmission, and tumor recurrence. Meanwhile, significant reductions in economic cost and the duration of hospitalization were shown in the ambulatory group than the conventional group, which was consistent with the main results. The results of DASS-21 were also consistent with the main results. Notably, though with no significance, the complication incidence of the ambulatory group was 12.9% higher than the conventional group among patients with thyroid cancer.

**Table 5:** Subgroup analyses among patients with thyroid cancer.

Characteristic	Ambulatory group (n=125)	Conventional group (n=81)	*P*
Duration of surgery (min)	193.21 ± 30.44	187.04 ± 28.92	0.149
Blood loss (ml)	19.27 ± 11.07	18.25 ± 8.15	0.446
Postoperative complications	47 (37.6)	20 (24.7)	0.068
Transient hypoparathyroidism	39 (24.5)	17 (21.0)	
Permanent hypoparathyroidism	4 (2.5)	0 (0.0)	
Hypoglycemia	0 (0.0)	0 (0.0)	
Transient voice change	3 (2.4)	2 (2.0)	
Seroma	1 (0.6)	0 (0.0)	
Pulmonary infection	0 (0.0)	1 (1.0)	
Hematoma	1 (0.6)	0 (0.0)	
Transfer to the conventional ward	2 (1.6)	–	–
30-days readmission	1 (0.8)	0 (0.0)	1.000
Recurrence of malignancy	1 (0.8)	1 (1.2)	0.633
Economic cost (yuan)	14778.09 ± 2092.45	20568.27 ± 4476.33	<0.001
Duration of hospitalization (day)	1.04 ± 0.32	5.54 ± 1.50	<0.001
Depression	3.82 ± 2.03	4.00 ± 1.83	0.528
Anxiety	7.92 ± 2.52	7.07 ± 2.65	0.022
Stress	6.95 ± 2.68	6.04 ± 2.91	0.022

Data are expressed as mean ± standard deviation or n (%).

## Discussion

For the very first time, this study summarized the data of patients undergoing ambulatory or conventional ETCBA in our medical center, and evaluated the safety, economic benefits, and mental health of patients, which showed that ambulatory ETCBA was as safe as the conventional ETCBA, but with great economic benefits and shorter duration of hospitalization regardless of patients with thyroid nodules or thyroid cancer. The results also highlighted that extra attention should be paid to manage the anxiety and stress of patients who received ambulatory ETCBA.

Many studies have proven the safety of ambulatory open thyroidectomy. A meta-analysis by Lee et al. consisting of 10 observational studies with 3427 patients suggested identical safety of outpatient thyroidectomy compared with inpatient thyroidectomy (11). More recently, a propensity score-matched study indicated outpatient thyroidectomy was not associated with a higher rate of readmission or complications (8). Furthermore, the safety of endoscopic thyroidectomy, including ETCBA, was well studied compared with open thyroidectomy. Jiang et al. included 20 publications with 5664 patients to perform a meta-analysis, and the results showed endoscopic thyroidectomy was similar to open thyroidectomy in terms of safety (12). This study stepped further to firstly investigate the safety of ambulatory ETCBA, which may provide the evidence for the adoption and development of ambulatory ETCBA. Meantime, the present study also showed significant economic benefits and a shorter duration of hospitalization in ambulatory ETCBA, which was solid but not surprising because the ambulatory management was administrated to achieve these purposes. Additionally, multiple studies on ambulatory surgery reported similar patient benefits (7, 13–16).

We noticed that included patients were mainly female, which might be caused by the low-level of aesthetic demands of male patients and could be noticed in papers focusing on endoscopic thyroid surgery as well (10, 17). Rare complications, such as seroma and hematoma, all occurred in-hospital within 24 hours after ETCBA, and did not need an invasive treatment or re-operation. However, though with no significance, the present study did show a higher complication rate of ambulatory ETCBA than conventional ETCBA (28.3% vs. 21.8%). Among patients with thyroid cancer, the complication incidence of the ambulatory group was even 12.9% higher than the conventional group. Thus, a prospective study with a larger cohort is needed to validate the safety of ambulatory ETCBA, especially among patients with thyroid cancer, and to investigate the reasons of it.

Nevertheless, this study also had some limitations. Firstly, we failed to investigate the role of ambulatory ETCBA due to the ambulatory ETCBA was currently not allowed in most medical centers. However we considered this study could serve as an important foundation from the conventional ETCBA to outpatient ETCBA. Secondly, patients with permanent hypoparathyroidism still had a chance to recover even after six months, which might cause inaccurate complication data. Thirdly, the follow-up time was too short to fully investigate the long-term outcomes, which would be focused on in the future report with continuous follow-up. Lastly, this study preliminarily showed the mental health assessment of patients undergone ETCBA. However, although no patient was reported to have a history of psychotropic drugs, the correlation may exist between the mental health and the diagnosis of disease. We failed to investigate it because the mental changes may happen before the diagnosis of thyroid nodules, and it is hard to access patients for such assessments before the diagnosis. Future studies will be performed to deal these questions for a more comprehensive assessment of patient mental health.

## Conclusion

Ambulatory ETCBA is as safe as conventional ETCBA for selective patients with thyroid nodules or thyroid cancer, however with significant economic benefits and shorter duration of hospitalization. Extra attention should be paid to manage the anxiety and stress of patients who received ambulatory ETCBA.

## Data Availability Statement

The raw data supporting the conclusions of this article will be made available by the authors, without undue reservation.

## Ethics Statement

The studies involving human participants were reviewed and approved by Institutional Ethics Committee of Xiangya Hospital, Central South University. Written informed consent for participation was not required for this study in accordance with the national legislation and the institutional requirements.

## Author Contributions

All authors made substantive intellectual contributions to this study to qualify as authors. XL conceived of the design of the study. FX modified the design of the study. ZZ and FX performed the study, collected the data, and contributed to the design of the study. ZZ analyzed the data. ZZ drafted Result, Discussion, and Conclusion sections. FX drafted Methods sections. FX and XL edited the manuscript. All authors read and approved the final manuscript. All authors have agreed to be accountable for all aspects of the work in ensuring that questions related to the accuracy or integrity of any part of the work are appropriately investigated and resolved.

## Funding

This work was supported by the National Natural Science Foundation of China (grant No. 82073262) and the Hunan Province Natural Science Foundation (grant number 2019JJ40475).

## Conflict of Interest

The authors declare that the research was conducted in the absence of any commercial or financial relationships that could be construed as a potential conflict of interest.

## Publisher’s Note

All claims expressed in this article are solely those of the authors and do not necessarily represent those of their affiliated organizations, or those of the publisher, the editors and the reviewers. Any product that may be evaluated in this article, or claim that may be made by its manufacturer, is not guaranteed or endorsed by the publisher.

## References

[1] Singh Ospina N Maraka S Espinosa De Ycaza AE Ahn HS Castro MR Morris JC. Physical Exam in Asymptomatic People Drivers the Detection of Thyroid Nodules Undergoing Ultrasound Guided Fine Needle Aspiration Biopsy. Endocrine (2016) 54(2):433–9. doi: 10.1007/s12020-016-1054-y PMC5584598 27510173

[2] Valderrabano P Khazai L Thompson ZJ Leon ME Otto KJ Hallanger-Johnson JE. Cancer Risk Stratification of Indeterminate Thyroid Nodules: A Cytological Approach. Thyroid: Off J Am Thyroid Assoc (2017) 27(10):1277–84. doi: 10.1089/thy.2017.0221 PMC6112164 28806881

[3] Wang C Feng Z Li J Yang W Zhai H Choi N. Endoscopic Thyroidectomy *via* Areola Approach: Summary of 1,250 Cases in a Single Institution. Surg Endoscopy (2015) 29(1):192–201. doi: 10.1007/s00464-014-3658-8 24986013

[4] Park KN Jung C Mok JO Kwak JJ Lee SW. Prospective Comparative Study of Endoscopic *via* Unilateral Axillobreast Approach Versus Open Conventional Total Thyroidectomy in Patients With Papillary Thyroid Carcinoma. Surg Endoscopy (2016) 30(9):3797–801. doi: 10.1007/s00464-015-4676-x 26659230

[5] Wu G Fu J Lin F Luo Y Lin E Yan W. Endoscopic Central Lymph Node Dissection *via* Breast Combined With Oral Approach for Papillary Thyroid Carcinoma: A Preliminary Study. World J Surg (2017) 41(9):2280–2. doi: 10.1007/s00268-017-4015-6 28417186

[6] Chai YJ Chung JK Anuwong A Dionigi G Kim HY Hwang KT. Transoral Endoscopic Thyroidectomy for Papillary Thyroid Microcarcinoma: Initial Experience of a Single Surgeon. Ann Surg Treat Res (2017) 93(2):70–5. doi: 10.4174/astr.2017.93.2.70 PMC5566749 28835882

[7] Zhang Z Xia F Wang W Jiang B Yao L Huang Y. Ambulatory Thyroidectomy is Safe and Beneficial in Papillary Thyroid Carcinoma: Randomized Controlled Trial. Head Neck (2020) 43(4):1116–21. doi: 10.1002/hed.26557 33247492

[8] Hu QL Livhits MJ Ko CY Yeh MW. Same-Day Discharge Is Not Associated With Increased Readmissions or Complications After Thyroid Operations. Surgery (2020) 167(1):117–23. doi: 10.1016/j.surg.2019.06.054 31582306

[9] Prabhakar A Helander E Chopra N Kaye AJ Urman RD Kaye AD. Preoperative Assessment for Ambulatory Surgery. Curr Pain Headache R (2017) 21(10):43. doi: 10.1007/s11916-017-0643-7 28861722

[10] Qu R Li J Yang J Sun P Gong J Wang C. Treatment of Differentiated Thyroid Cancer: Can Endoscopic Thyroidectomy *via* a Chest-Breast Approach Achieve Similar Therapeutic Effects as Open Surgery? Surg Endoscopy (2018) 32(12):4749–56. doi: 10.1007/s00464-018-6221-1 29761277

[11] Lee DJ Chin CJ Hong CJ Perera S Witterick IJ. Outpatient Versus Inpatient Thyroidectomy: A Systematic Review and Meta-Analysis. Head Neck (2018) 40(1):192–202. doi: 10.1002/hed.24934 29120517

[12] Jiang W Yan PJ Zhao CL Si MB Tian W Zhang YJ. Comparison of Total Endoscopic Thyroidectomy With Conventional Open Thyroidectomy for Treatment of Papillary Thyroid Cancer: A Systematic Review and Meta-Analysis. Surg Endoscopy (2020) 34(5):1891–903. doi: 10.1007/s00464-019-07283-y 32144555

[13] Nordin AB Shah SR Kenney BD. Ambulatory Pediatric Surgery. Semin Pediatr Surg (2018) 27(2):75–8. doi: 10.1053/j.sempedsurg.2018.02.003 29548355

[14] Thompson NB Calandruccio JH. Hand Surgery in the Ambulatory Surgery Center. Orthopedic Clinics North America (2018) 49(1):69–72. doi: 10.1016/j.ocl.2017.08.009 29145986

[15] Rider CM Hong VY Westbrooks TJ Wang J Sheffer BW Kelly DM. Surgical Treatment of Supracondylar Humeral Fractures in a Freestanding Ambulatory Surgery Center is as Safe as and Faster and More Cost-Effective Than in a Children’s Hospital. J Pediatr Orthopedics (2018) 38(6):e343–8. doi: 10.1097/BPO.0000000000001171 29664879

[16] Ford MC Walters JD Mulligan RP Dabov GD Mihalko WM Mascioli AM. Safety and Cost-Effectiveness of Outpatient Unicompartmental Knee Arthroplasty in the Ambulatory Surgery Center: A Matched Cohort Study. Orthopedic Clinics North Am (2020) 51(1):1–5. doi: 10.1016/j.ocl.2019.08.001 31739873

[17] Guo Y Qu R Huo J Wang C Hu X Chen C. Technique for Endoscopic Thyroidectomy With Selective Lateral Neck Dissection *via* a Chest-Breast Approach. Surg Endoscopy (2019) 33(4):1334–41. doi: 10.1007/s00464-018-06608-7 30569419

