# Supplementary material for: Ambulatory Endoscopic Thyroidectomy via a Chest-Breast Approach Has an Acceptable Safety Profile for Thyroid Nodule
Source: Front Endocrinol (Lausanne). 2021 Dec 20;12:795627. doi: 10.3389/fendo.2021.795627 (PMC8721221; doi:10.3389/fendo.2021.795627)
Supplement: Supplementary file 1 [file Table_1.docx]

**Questionnaire of Depression Anxiety Stress Scales-21 (DASS-21)**

| Question | Score | | | |
| --- | --- | --- | --- | --- |
| 1. I found it hard to wind down. | 0 | 1 | 2 | 3 |
| 1. I was aware of dryness of my mouth. | 0 | 1 | 2 | 3 |
| 1. I couldn’t seem experience any positive feeling at all. | 0 | 1 | 2 | 3 |
| 1. I experienced breathing difficulty. | 0 | 1 | 2 | 3 |
| 1. I found it difficult to work up the initiative to do things | 0 | 1 | 2 | 3 |
| 1. I tended to over-react to situations. | 0 | 1 | 2 | 3 |
| 1. I experienced trembling. | 0 | 1 | 2 | 3 |
| 1. I felt that i was using a lot of nervous energy. | 0 | 1 | 2 | 3 |
| 1. I was worried about situations in which i might panic and make a fool of myself. | 0 | 1 | 2 | 3 |
| 1. I felt that i had nothing to look forward to. | 0 | 1 | 2 | 3 |
| 1. I found myself getting agitated. | 0 | 1 | 2 | 3 |
| 1. I found it difficult to relax. | 0 | 1 | 2 | 3 |
| 1. I felt downhearted and blue. | 0 | 1 | 2 | 3 |
| 1. I was intolerant of anything that kept me from getting on with what i was doing. | 0 | 1 | 2 | 3 |
| 1. I felt i was close to panic. | 0 | 1 | 2 | 3 |
| 1. I was unable to become enthusiastic about anything. | 0 | 1 | 2 | 3 |
| 1. I felt that i wasn’t worth much as a person. | 0 | 1 | 2 | 3 |
| 1. I felt i was rather touchy. | 0 | 1 | 2 | 3 |
| 1. I was aware of the action of my heart in the absence of physical exertion. | 0 | 1 | 2 | 3 |
| 1. I felt scared without any good reason. | 0 | 1 | 2 | 3 |
| 1. I felt that life was meaning less. | 0 | 1 | 2 | 3 |

Rating scale：

0 - Did not apply to me at all

1 - Applied to me some degree, or some of the time

2 - Applied to me a considerable degree, or a good part of time

3 - Applied to me very much, or most of the time

Item 1, 6, 8, 11, 12, 14, 18 belong to Stress factor.

Item 2, 4, 7, 9, 15, 19, 20 belong to Anxiety factor.

Item 3, 5, 10, 13, 16, 17, 21 belong to Depression factor.

Reference:

Lovibond, P. F., & Lovibond, S. H. (1995). The structure of negative emotional states: Comparison of the Depression Anxiety Stress Scales (DASS) with the Beck Depression and Anxiety Inventories. Behaviour Research and Therapy, 33, 335–343.

Antony, M. M., Bieling, P. J., Cox, B. J., Enns, M. W., & Swinson, R. P. (1998). Psychometric properties of the 42-item and 21-item versions of the depression anxiety stress scales in clinical groups and a community sample. Psychological Assessment, 10, 176–181.
